# Evaluating Leaf and Canopy Reflectance of Stressed Rice Plants to Monitor Arsenic Contamination

**DOI:** 10.3390/ijerph13060606

**Published:** 2016-06-18

**Authors:** Varaprasad Bandaru, Craig S. Daughtry, Eton E. Codling, David J. Hansen, Susan White-Hansen, Carrie E. Green

**Affiliations:** 1 Department of Geographical Sciences, University of Maryland, College Park, MD 20740, USA; 2 USDA-ARS Hydrology and Remote Sensing Laboratory, Beltsville, MD 20705, USA; Craig.Daughtry@ars.usda.gov; 3 USDA-ARS Crop Systems and Global Change Laboratory, Beltsville, MD 20705, USA; eton.codling@ars.usda.gov (E.E.C.); carrie.green@ars.usda.gov (C.E.G.); 4 Outreach & Engagement/Extension Administration, Oregon State University, Corvallis, OR 97331, USA; david.hansen@oregonstate.edu; 5 Department of Horticulture, Oregon State University, Corvallis, OR 97331, USA; susan.white@oregonstate.edu

**Keywords:** plant stress, leaf chlorophyll, rice, arsenic uptake, spectral reflectance, SAIL model, red edge, vegetative indices, LAI, soil reflectance

## Abstract

Arsenic contamination is a serious problem in rice cultivated soils of many developing countries. Hence, it is critical to monitor and control arsenic uptake in rice plants to avoid adverse effects on human health. This study evaluated the feasibility of using reflectance spectroscopy to monitor arsenic in rice plants. Four arsenic levels were induced in hydroponically grown rice plants with application of 0, 5, 10 and 20 µmol·L^−1^ sodium arsenate. Reflectance spectra of upper fully expanded leaves were acquired over visible and infrared (NIR) wavelengths. Additionally, canopy reflectance for the four arsenic levels was simulated using SAIL (Scattering by Arbitrarily Inclined Leaves) model for various soil moisture conditions and leaf area indices (LAI). Further, sensitivity of various vegetative indices (VIs) to arsenic levels was assessed. Results suggest that plants accumulate high arsenic amounts causing plant stress and changes in reflectance characteristics. All leaf spectra based VIs related strongly with arsenic with coefficient of determination (r^2^) greater than 0.6 while at canopy scale, background reflectance and LAI confounded with spectral signals of arsenic affecting the VIs’ performance. Among studied VIs, combined index, transformed chlorophyll absorption reflectance index (TCARI)/optimized soil adjusted vegetation index (OSAVI) exhibited higher sensitivity to arsenic levels and better resistance to soil backgrounds and LAI followed by red edge based VIs (modified chlorophyll absorption reflectance index (MCARI) and TCARI) suggesting that these VIs could prove to be valuable aids for monitoring arsenic in rice fields.

## 1. Introduction

Arsenic (As) contamination in rice (*Oriza sativa* L.) is a critical issue in many developing countries, especially Bangladesh, India and China [1]. Major sources of As in rice cultivation include contaminated irrigation water and elevated As in paddy fields from metal mining [2]. Under such conditions, As (as arsenate) is likely transported to aboveground plant parts as a phosphate analog [3,4,5]. The United Nations world health organization recommends a statutory limit of 0.2–0.4 mg·kg^−1^ As in rice grains [6], earlier studies reported levels as high as 1.8 mg·kg^−1^ in rice grains [7]. These levels of As can result in potentially dangerous As ingestion by humans and animals [8] and cause serious health effects.

Given the concerns of As in rice, numerous research efforts have been conducted to assess the As contamination in paddy soils [9,10,11,12], and to develop mitigation strategies to reduce the As absorption into rice plants [13,14,15,16]. Regular field sampling followed by wet chemistry methods and interpolation techniques is a common method for detecting As contamination [17,18], which is highly tedious and expensive. In addition, when mitigation strategies (e.g., silicon fertilization) are implemented to control As uptake into plant parts, it is essential to monitor degree of reduction regularly to ensure complete mitigation of As accumulation. It is highly challenging to use the field sampling and wet chemistry methods for regular monitoring of As uptake at large scales. Therefore, research that focuses on developing large scale mapping and monitoring methods are essential.

In plants, As accumulation can generate reactive oxygen species which can directly affect metabolic functions such as cell division and photosynthesis [19,20,21]. Recent studies observed dramatic reductions in chlorophyll content, stunted plant growth, and chlorotic symptoms with As accumulation in rice plants [22,23,24]. The plants displaying these symptoms are typically considered as stressed plants.

Hyperspectral remote sensing has been explored for developing methods to assess and monitor plant stress caused by numerous factors (e.g., nutrient and water deficiency, diseases, metal accumulation) [25,26,27,28]. Changes in leaf biochemical contents (e.g., chlorophyll) with plant stress affect plant spectral properties (reflectance and transmittance) [29] at specific wave lengths (e.g., red, green, blue and red edge bands). Using these changes, predictive models have been developed to assess plant stress using various statistical approaches (e.g., linear regression and partial least-squares regression (PLSR) and random forests) [25,30]. Extraneous factors (e.g., soil background (BG) reflectance) often confound with spectral response to the variable of interest, and to reduce their impact, two or more bands are used to develop vegetative indices (VIs) using ratios, slopes or other formulations [31]. The VIs vary in their performance depending on the degree of resistance to parameters like leaf area per unit ground surface area known as Leaf Area Index (LAI) and soil background reflectance [32]. Therefore, when selecting VI, it is important to evaluate the VIs for their sensitivity to the differences in plant stress as well as for their resistance to extraneous factors.

Given the aforementioned information, it might be feasible to monitor As levels in paddy fields using changes in spectral reflectance properties due to As-induced plant stress. The main objective of this study was to evaluate the effectiveness of visible and near infrared spectral reflectance at leaf and canopy scales to quantify the As levels in rice plants. Specific objectives are: (1) to understand the effects of As uptake in rice on leaf and canopy reflectance; (2) to evaluate the performance of different hyperspectral vegetation indices at both leaf and canopy scales to monitor As levels in rice plants.

## 2. Materials and Methods

### 2.1. Hydroponic Growth Chamber Experiment

An experiment was conducted under controlled conditions in a growth chamber at the USDA Beltsville Agricultural Research Facility in Beltsville, MD. The experiment was arranged as a completely randomized design with four treatments and five replications. Treatments consisted of one control and three levels of As (added as Na_2_HAsO_4_): 5, 10, and 20 μmol·As·L^−1^. Treatments are designated as control, low, medium and high based on soluble As concentration.

**Figure 1:**
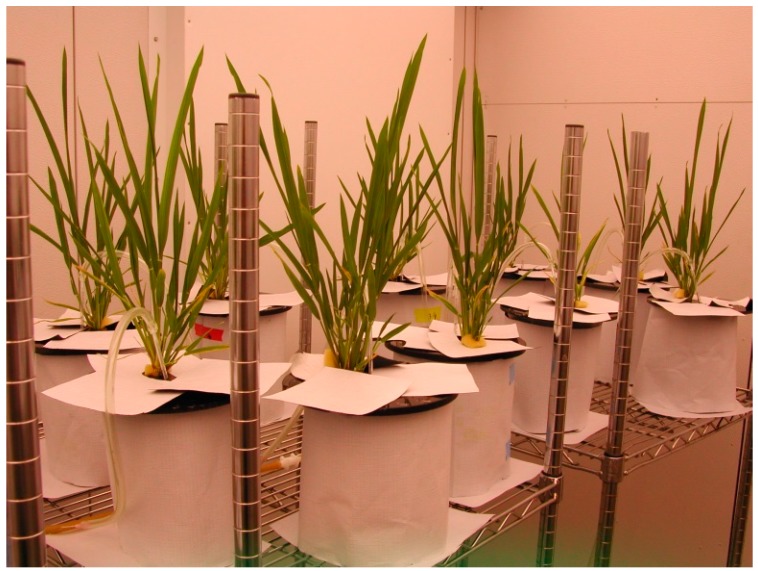
Rice plants under different arsenic treatments growing hydroponically in an aerated standing nutrient solution.

Rice seeds of the Jefferson cultivar were germinated on standard germination paper saturated with a modified Hoagland solution (2.0 mM Ca(NO_3_)_2_: KNO_3_, 0.8 mM MgSO_4_, and 0.8 mM K_2_HPO_4_) [33]. Five days after germination, seedlings were transferred to 2.5 L polyethylene beakers; each beaker contained three seedlings grouped into one bundle supported by polyurethane foam (Figure 1). After seedlings were transferred, the As treatments were imposed with a modified Hoagland nutrient solution. The hydroponic system used for this experiment was an aerated standing-nutrient solution [33]. The composition of nutrient solution is presented in Table 1. Deionized water was added to each bucket every other day to maintain a constant volume of the solution in each beaker. The nutrient solution in each beaker was completely replaced once per week. 

Seedlings were exposed to a photosynthetic photon flux density (PPFD) of 300 μmol·m^−2^·s^−1^ provided by a combination of fluorescent tubes and incandescent bulbs for 16 h each day resulting in a daily integrated photon flux of 17.3 mol·m^−2^. Temperature was maintained at 26 °C/20 °C day/night at a relative humidity of 70%–80%. Solution pH was monitored and adjusted every other day to maintain a pH of 6.0 (5.5 to 6.5). After two weeks, 10 μM of nitrogen (as NH_4_NO_3_) and phosphorus (as K_2_HPO_4_) were supplemented every two days. Plants were grown for approximately eight weeks; until mid- to late-tillering stage (V7–V8). During the last three weeks before harvesting, supplemented solution concentrations of nitrogen and phosphorus were raised to 200 μM and 20 μM, respectively. 

### 2.2. Measurement Procedures

#### 2.2.1. Leaf Spectral Measurements

At mid- to late-tillering stage (V7–V8), four fully-expanded leaves near the top of each bundle were excised for spectral reflectance measurement. Leaf reflectance and transmittance was measured with an integrating sphere (LiCor LI-1800, Lincoln, NE) coupled with a fiber optic cableprobe to an ASD FieldSpec^®^ Spectroradiometer (Analytical Spectral Devices, Inc., Boulder, CO, USA) [34]. Reflectance was measured across the 400 to 2500 nm wavelength range at 1 nm resolution, and leaf reflectance factors were calculated using equations by Daughtry *et al.* [31]. Further, data were evaluated for normality using univariate analysis (SAS). Reflectance values were deleted if the absolute value of kurtosis or skewness was greater than 1.5.

#### 2.2.2. Bio-Physicochemical Measurements

After leaf reflectance was measured, two leaf disks (0.64 cm^2^) were punched from the leaf portion for which optical properties were measured. Disks were placed immediately into 3.5 mL dimethyl sulfoxide and kept in the dark at room temperature for 24 h to allow pigment extraction. Absorption measurements of the pigment extracts were made at 1 nm resolution using a dual-beam spectrophotometer (Perkin-Elmer, Wellesley, MA, USA). Chlorophyll concentrations were calculated using equations described by Wellburn [35]. Later, leaves of each treatment on which reflectance was measured were placed in individual paper bags, oven-dried at 65 °C to constant weight, and dry weights recorded. Plant materials were ashed and acid digested with concentrated trace-element grade HNO_3_ [33]. Leaf As concentrations were determined by inductively coupled plasma atomic emission spectrometry (ICP-AES) using the hydride generation method described by Codling and Ritchie [36]. Two National Institute of Standards and Technology (NIST) standard reference samples and two blanks were included for quality control for every 24 samples digested. Prior to analysis, all glassware and plasticware were acid washed in 3.0 N H_2_SO_4_, rinsed in de-ionized water, and air-dried.

#### 2.2.3. Soil Reflectance Measurements

Soil reflectance spectra for three moisture conditions were measured. Although rice is typically grown under submerged soil condition, direct seeding is also a common cultivation practice where both dry and wet soil conditions can be observed. Therefore, three soil conditions including dry, wet and submerged soils were used in this study.

DeWitt silt loam (Fine, smectitic, thermic Typic Albaqualfs) soil was collected from a rice research field near Stuttgart, Arkansas, USA. The soil was oven-dried at 105 °C and was then ground to pass a 2-mm screen. Subsequently, 800 g of soil was placed in sample trays (24.5 cm diameter × 8.5 cm deep) that were painted flat black. Water was added to each tray to produce three soil moisture conditions, 0, 0.4, and 2.5 g water g^−1^ soil. Water covered the soil in the highest treatment to a depth of approximately 5 cm to impose submerged soil condition. Triplicate trays were prepared. The sample trays were covered and allowed to equilibrate for 24 h before reflectance measurements. Reflectance spectra were acquired with a spectroradiometer (FieldSpec FS3, Analytical Spectral Devices, Boulder, CO, USA) over the 350 to 2500 nm wavelength region at 1-nm intervals. The samples were illuminated by six 100-W quartz-halogen lamps mounted on the arms of a camera copy stand at 55 cm over the sample at a 30° illumination zenith angle. A current-regulated DC power supply stabilized the output of the lamps. The 10° fore-optic of the spectroradiometer was aligned and positioned 50 cm from the sample surface at a 0° view zenith angle. The diameter of the field of view of the spectroradiometer was 8.5 cm. The illumination and view angles were chosen to minimize shadowing and to emphasize the fundamental spectral properties of the samples. Four spectra of 30 scans each were acquired from each sample by rotating the sample tray 90° after each spectrum. A 30-cm square Spectralon (Labsphere, Inc., North Sutton, NH, USA) reference panel was placed in the field of view and was illuminated and viewed in the same manner as the samples. Reflectance factors were calculated and corrected for the reflectance of the Spectralon reference panel [37]. 

### 2.3. Simulated Canopy Reflectance

Canopy reflectance was simulated using the Scattering by Arbitrarily Inclined Leaves (SAIL) model, a turbid-medium model that considers canopy as horizontally uniform plane having infinitely extended medium with diffusely reflecting and transmitting elements [38]. The model inputs include leaf and background spectral data, viewing and illumination parameters, LAI values, and leaf angle distribution functions. Mean leaf reflectance and transmittance of four As treatments, and mean soil reflectance under three moisture conditions were used as input spectral data. Details of other input conditions are listed in Table 2. Canopy reflectance factors were simulated for eight LAI (0, 0.1, 0.5, 1.0, 1.5, 2.0, 4.0, 6.0) treatments and three soil moisture conditions.

### 2.4. Vegetative Indices

Earlier studies explored numerous vegetative indices for assessing changes in leaf structure and pigment concentration as a function of different plant stress factors [39,40,41,42,43]. Most of these vegetative indices are primarily based on combinations of two or more reflectance factors at blue, green, red, NIR and red edge bands. Leaf pigments strongly absorb radiation at the red and blue bands and therefore these regions become relatively insensitive to changes in leaf pigments as LAI increases [44]. Conversely, green and red edge regions are very sensitive to subtle change in plant stress as leaf pigments reflect radiation strongly at these regions [44].

In this study, measured leaf reflectance and simulated canopy reflectance at different narrow bands were used to determine five different vegetative indices at leaf and canopy scales, respectively. Narrow bands were computed by aggregating spectral reflectance factors over 10 nm wide wavelength ranges: blue band (Rb)—475–485 nm, green band (Rg)—545–555 nm, red band (Rr)—665–675 nm, red edge band (Re)—715–725 nm, and near infrared band (Rn)—845–855 nm. Rr and Rn bands were used to compute normalized difference vegetative index (NDVI) and optimized soil adjusted vegetation index (OSAVI), while Re band in combination with Rr and Rg bands were used to compute modified chlorophyll absorption reflectance index (MCARI) and transformed chlorophyll absorption reflectance index (TCARI). OSAVI and TCARI were used to determine TCARI/OSAVI combined index.

In addition to reflectance based indices, derivative reflectance was used to determine peak derivative ratio [45]. Derivative reflectance is expected to reduce the impacts of background noise on the spectral information of the target features, and therefore it has been used in the place of spectral reflectance. Smith *et al.* [45] found double peaks in the red edge region and used the ratio of 1st derivative reflectance at double peak wavelengths (725 and 702 nm) for detecting plant stress caused by gas leaks. Similar to Smith *et al.* [45], here we analyzed 1st derivative reflectance to identify that the double peak in the red edge region resulted from different As treatments, and subsequently computed double peak derivative ratio for different As treatments at leaf and canopy scales. Hereafter, this ratio is referred to as peak derivative ratio (PDR) for conciseness. The details of studied indices are listed in Table 3.

### 2.5. Data Analyses

Measured variables, such as total As in plant tissue, leaf chlorophyll, and plant dry weights, were tested using the General Linear Models, ANOVA procedure from the Statistical Analysis System software [38]. When main effects were significant, treatment means for dependent plant characteristics were separated using a protected LSD separation (α = 0.05). Performance of the vegetative indices at leaf level was evaluated using regression analysis.

## 3. Results and Discussion

### 3.1. Plant as Uptake and Plant Stress

**Figure 2:**
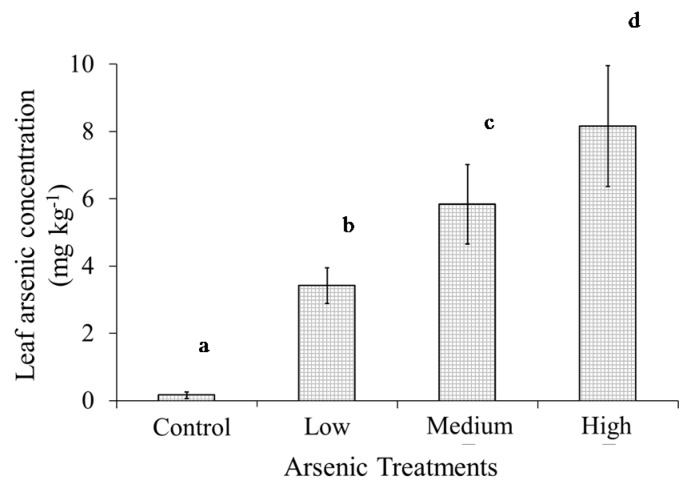
Mean (number of observations, *n* = 20) accumulated As concentration under control, low, medium and high As treatments. Means with different letters are significantly different at probability (α) of 0.05. Error bars represent standard deviations.

**Figure 3:**
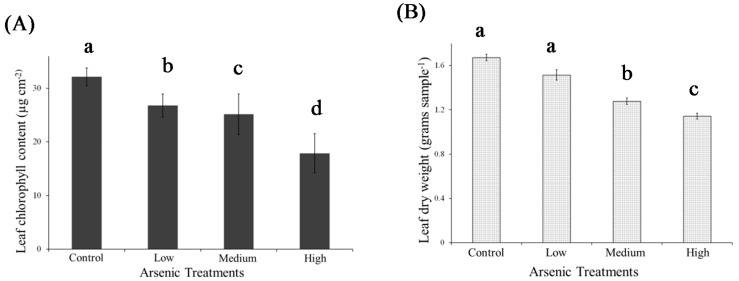
Mean (number of observations, *n* = 20) leaf chlorophyll content (**A**) and leaf dry weight (grams sample^−1^) (**B**) under control, low, medium and high As treatments. Means with different letters are significantly different at probability (α) of 0.05. Error bars represent standard deviations.

**Figure 4:**
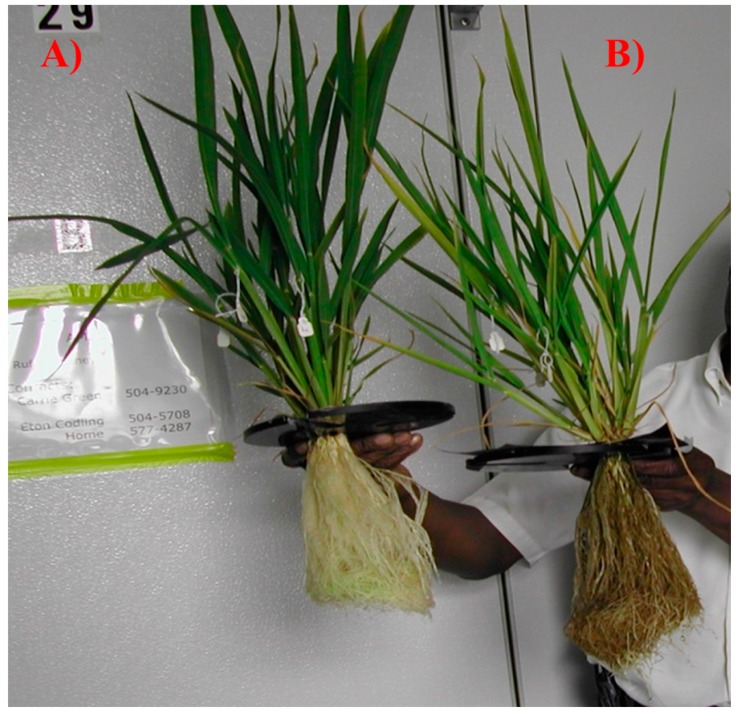
Visual difference between hydroponically grown rice under control (**A**) and high As (**B**) treatments.

The leaf As concentration increased significantly (*p* < 0.01), ranging from 0 mg·kg^−1^ in the control to 8.13 mg·kg^−1^ in the high As treatment (Figure 2). Similar trends of As uptake in rice have been reported [24]. The accumulation of arsenic induced alterations in plant biophysical and biochemical characteristics was indicated by significant changes (*p* < 0.01) in plant dry mass and leaf chlorophyll content (Figure 3). Results showed that compared to the control treatment, leaf dry matter decreased by 11.6%, 22.1%, and 30.7% in low, medium, and high As treatments, respectively, while total chlorophyll concentration decreased by 12.4%, 29.3% and 43.9%, respectively (Figure 3). Earlier studies [23,24] attributed these changes to damage of leaf chloroplast membrane structures associated with As toxicity. Further, changes in biophysical and biochemical characteristics were reflected in plant visible symptoms including reduced plant growth, chlorosis along the leaf margins, and reddish-brown discoloration of roots in As treated plants (Figure 4).

### 3.2. Soil Background Reflectance

**Figure 5:**
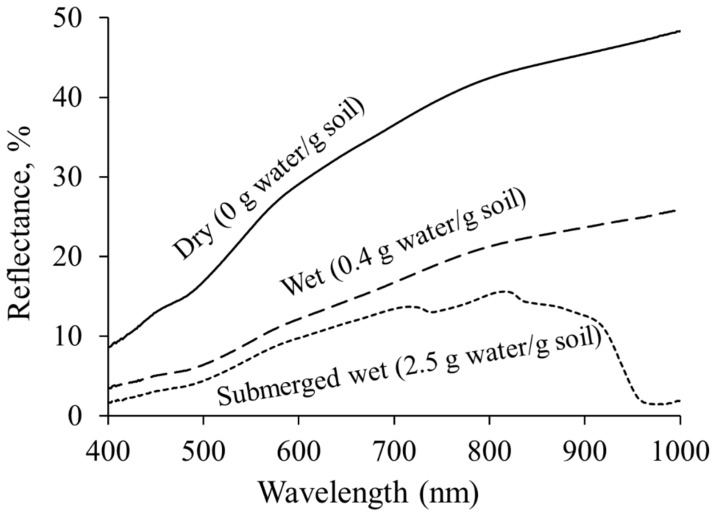
Reflectance of DeWitt silt loam soil under dry (0 g water g^−1^ soil), wet (0.2 gram water gram soil^−1^) and submerged wet conditions (2.5 gram water gram soil^−1^).

Reflectance decreased with increase in moisture content however the degree of response varied with wavelength (Figure 5). At Near Infrared (NIR) wavelengths, reflectance exhibited drastic decrease with change in moisture content whereas reflectance showed minor decrease at visible wavelengths. For instance, NIR reflectance for soil submerged was approximately 45%–92% less than for wet soil. Conversely soil reflectance at visible wavelengths was only 15%–25% less under submerged condition. The differences in effect of soil moisture at different wavelengths can be attributed to differences in water absorption characteristics. Visible wavelengths are less sensitive to water than near infrared wavelengths [49]. These results indicate that vegetative NIR reflectance is substantially affected by moist soil background, and therefore vegetative indices based on NIR reflectance may not be well suited for measuring vegetation status under moist soil background.

### 3.3. Leaf Reflectance and Derivative Reflectance

**Figure 6:**
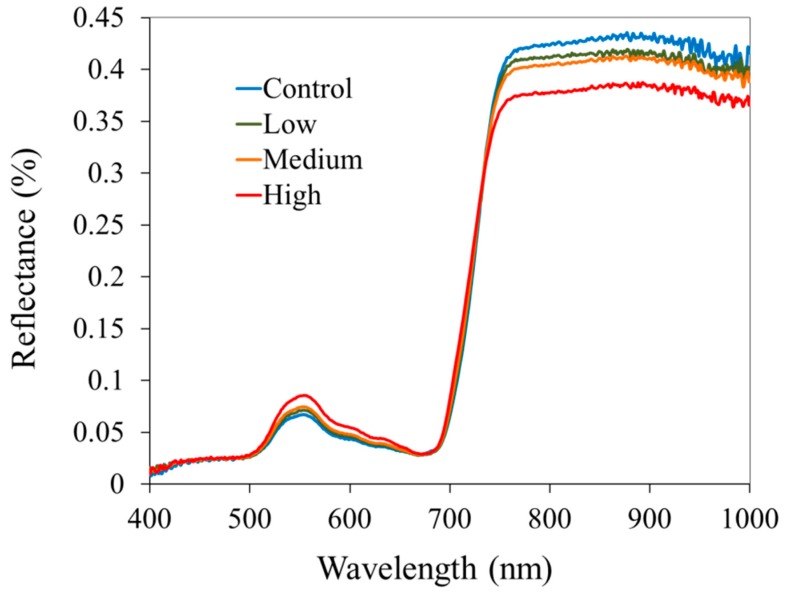
Mean leaf reflectance spectra of rice grown under four levels of arsenic. Leaves were sampled when the rice plants were at mid- to late-tillering stage (V7–V8).

As shown in Figure 6, leaf reflectance exhibited considerable changes with increased As accumulation. Leaf reflectance increased with As accumulation for visible wavelengths but decreased the NIR reflectance (at >750 nm). The observed reflectance behavior is a typical response to plant stress caused by other heavy metals and nitrogen deficiency noticed in earlier studies [50,51,52]. Decline in chlorophyll content due to plant stress induces low absorption of light and increase in leaf reflectance [24]. Increased NIR reflectance can be attributed to leaf structural changes which affected internal scattering of the light [27].

**Figure 7:**
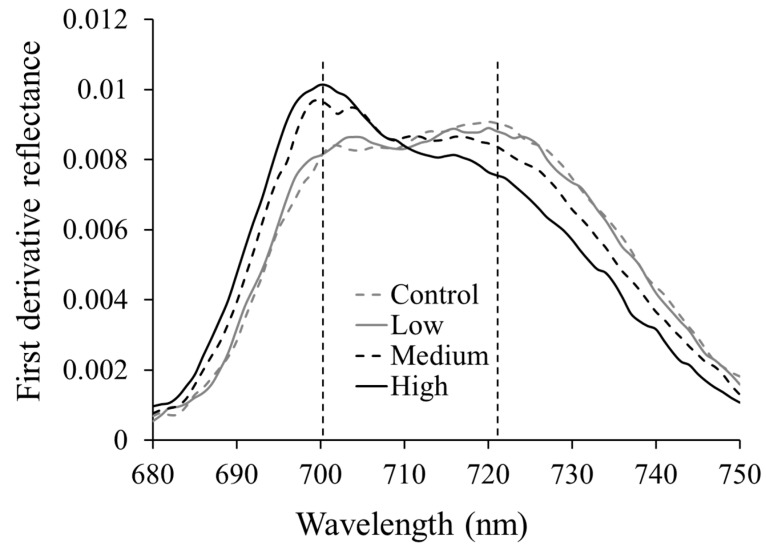
Mean derivative reflectance of rice plants under different As levels. Primary and secondary peak values were located around 720 nm and 700 nm.

First derivative reflectance calculations revealed a primary peak and a secondary peak in the red edge region (Figure 7). In the control treatment, the primary peak was located near 720 nm and the secondary peak was near 700 nm. The magnitude of the primary peak decreased while the magnitude of the secondary peak increased with increasing As treatment. Smith *et al.* [45] observed similar changes in peak positions at 702 and 725–730 nm while studying soil contamination with gas leaks in winter wheat, grass, and barley canopies. Similarly, Horler *et al.* [53] identified two peak positions around 700 and 725 nm in their study with zinc and copper contamination in different crops. They both attributed these changes to decreased leaf chlorophyll content with increasing soil contamination. Increased peak magnitude around 700 nm with As treatment could be caused by weak absorption of red light with decreased chlorophyll content. Low cellular differentiation and cell distortion due to changes in metabolic reactions could have increased intercellular scattering, resulting in a decreased peak at 720 nm. Using peak derivative reflectance values at 720 and 700 nm, peak derivative ratio was determined for further analysis.

### 3.4. Canopy Reflectance

**Figure 8:**
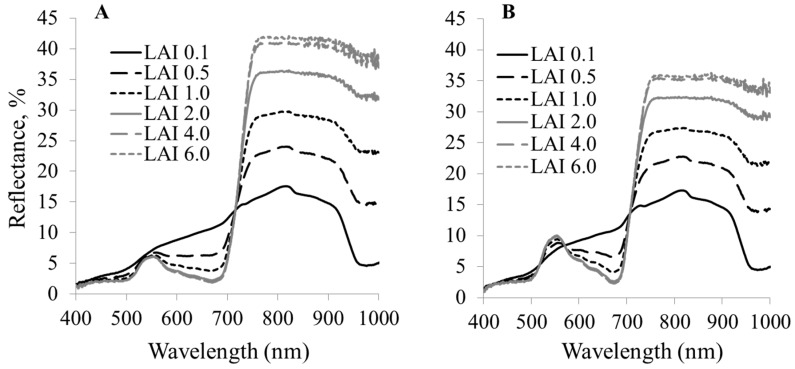
Simulated canopy reflectance spectra of (**A**) control (no As) and (**B**) high As on submerged wet soil.

**Figure 9:**
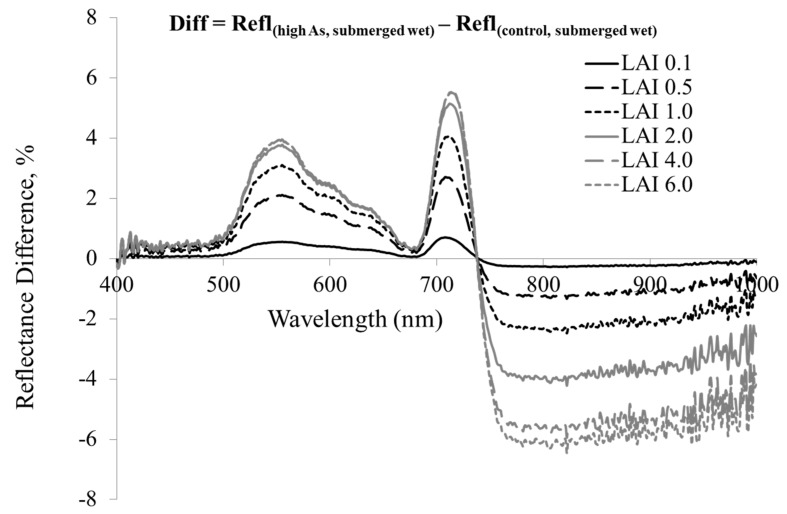
Scene difference reflectance spectra of rice for variable leaf optics (control *vs.* high As levels) under submerged soils. Difference = Reflectance (high As, submerged soil)—Reflectance (control, submerged soil).

Canopy reflectance is generally affected by many factors such as LAI and underlying soil conditions. As shown in Figure 8, the contribution of background reflectance to canopy reflectance declined substantially for LAI greater than 2. As such, canopy reflectance exhibited significant decrease under wet soil and submerged conditions. The variation in canopy reflectance with arsenic stress is shown in Figure 9. It is apparent that the difference in reflectance between plants under control and high As levels is higher at green and red edge wavelengths even at LAI value <2 indicating their higher sensitivity to the arsenic stress at canopy scale. It implies that vegetative indices based on green and red edge reflectance could have better linearity with As stress and higher resistance to soil reflectance for submerged soils.

### 3.5. Relationship between Vegetative Indices and Plant as Levels at Leaf and Canopy Scale

#### 3.5.1. Leaf Scale

The leaf vegetative indices exhibited strong positive relationship with leaf arsenic content with a coefficient of determination (r^2^) greater than 0.6 and RMSE values less than 2.0 (Table 4). The VIs based on red edge band showed stronger relationships with As content than traditional VIs based on NIR and red bands. Red edge band was more sensitive to leaf chlorophyll content affected with plant stress caused by various environmental factors and earlier studies demonstrated that VIs based on red edge performs better to indicate plant stress conditions [26,54,55]. Among studied VIs based on red edge bands, the combined index, TCARI/OSAVI yielded the highest r^2^ value of 0.89 with RMSE value of 1.12, followed by TCARI (r^2^ = 0.88 and RMSE = 1.10) MCARI (r^2^ = 0.85 and RMSE = 1.23) and derivative ratio (r^2^ = 0.79 and RMSE = 1.45) (Table 4). At leaf scale, the reflectance characteristics are least affected by extraneous factors which is not the case at canopy scale where many factors confound with reflectance signal indicating plant stress condition. Hence, VIs that performed well at leaf scale may not exhibit same relationships at canopy scale.

#### 3.5.2. Canopy Scale

**Figure 10:**
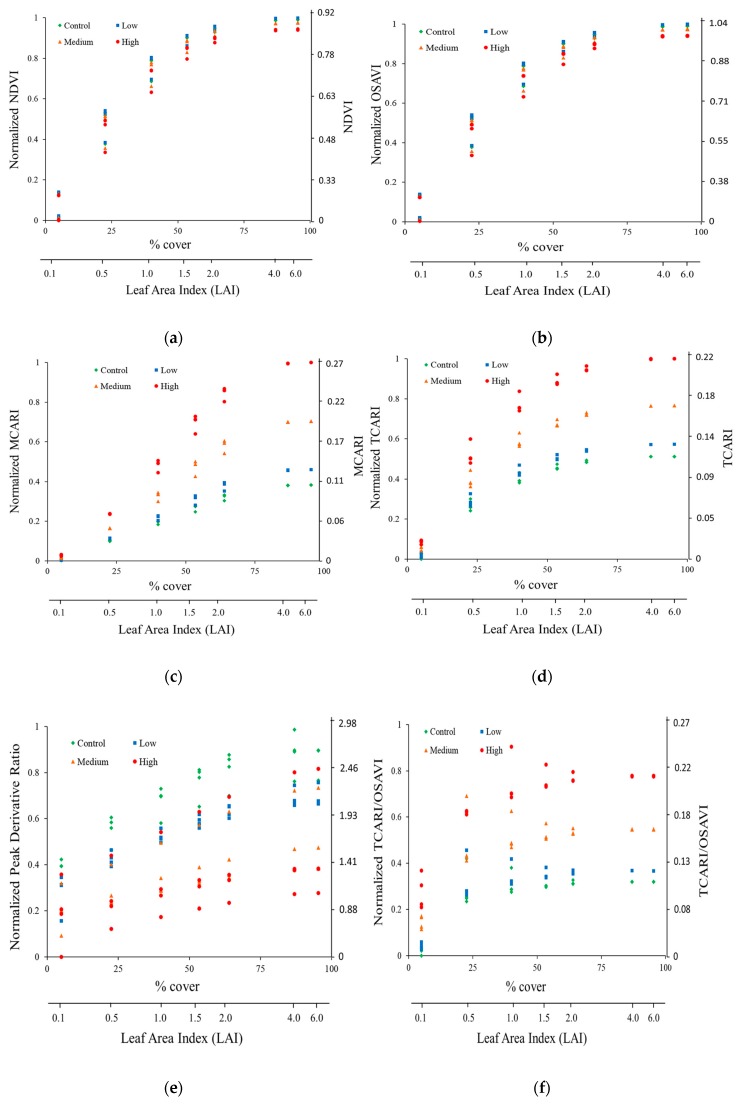
Simulated vegetative indices for four leaf arsenic levels and three soil moisture conditions as function of foliage cover (LAI). (**a**) NDVI (**b**) OSAVI (**c**) MCARI (**d**) TCARI (**e**) Peak derivative ratio (**f**) TCARI/OSAVI.

To understand the sensitivity of VIs at canopy scale, VIs for various soil backgrounds and leaf arsenic levels were compared as a function of LAI or foliage coverage (Figure 10). For ease of comparison, all VIs were normalized between 0 and 1, and foliage coverage was estimated (Equation (1)) to use in comparison along with LAI as VIs were found in earlier studies showing more linear relationship than LAI [31].

Foliage cover = 100 [1 − exp(−0.51 LAI)]
(1)

Traditional VIs based on red and NIR bands including NDVI and OSAVI were shown to be sensitive to foliage coverage or LAI and insensitive to soil background and arsenic levels. The range of NDVI and OSAVI values decreased as foliage coverage (or LAI) increased suggesting high sensitivity to foliage coverage or LAI values, and showed no clear separation for different arsenic levels and soil backgrounds indicating insensitivity to both soil background and leaf arsenic content. Similar results were reported in earlier studies focused on chlorophyll detection [31,32]. The MCARI and TCARI values exhibited clear separation for arsenic levels, and differences are more conspicuous at LAI > 2. The higher the values of MCARI and TCARI, the higher the arsenic content, and vice versa. The change in range of MCARI and TCARI values with increase in LAI indicates high sensitivity to LAI values. The Derivative ratio was sensitive to all three variables (*i.e.*, LAI, arsenic content and soil background) as values showed considerable separation of values from the beginning of LAI and no clear clustering of values for arsenic levels suggesting confounding effect of soil background reflectance and LAI or foliage cover. Combined index, TCARI/OSAVI showed pronounced clustering for arsenic levels even at lower LAI values. Eitle *et al.* [32] reported similar patterns with combined indices.

To confirm the trends observed in Figure 10, we performed analysis of variance (ANOVA) to assess the relative impact of soil background, LAI and arsenic content to the variations in VIs (Table 5). Results suggested that for NDVI and OSAVI, LAI accounted for approximately 98% of the variation, indicating high sensitivity of these indices to LAI and minimal sensitivity to arsenic levels and soils. For MCARI and TCARI, main effects of arsenic content and LAI accounted for >95% variation suggesting that these indices are sensitive to both LAI and arsenic, and insensitive to soil background. Although impact of LAI on peak derivative ratio is less comparative to TCARI and MCARI, soil background reflectance has substantially higher contribution to the derivative ratio indicating higher sensitivity to soil backgrounds. For the combined ratio, TCARI/OSAVI, arsenic levels accounted for 74% variation which suggests that TCARI/OSAVI can resist the interference of soil backgrounds and LAI better and perform well at canopy scale.

## 4. Conclusions

The main objective of this research was to evaluate the feasibility of using spectral characteristics to monitor arsenic levels in paddy rice crops. Hydroponic study indicated that rice plants can accumulate significant amount of arsenic into aboveground plant parts, and As can induce plant stress through affecting leaf chlorophyll concentration. Spectroscopic analysis suggested that arsenic-induced plant stress produced significant differences in leaf spectral characteristics which could be useful in monitoring arsenic levels in rice. All vegetative indices (NDVI, OSAVI, MCARI, TCARI, derivative ratio and TCARI/OSAVI) based on leaf spectra were strongly related with arsenic levels. However, at canopy scale, soil background reflectance and canopy cover obscure spectral signals of arsenic induced plant stress affecting the performance of VIs. Traditional VIs based on red and NIR bands were highly sensitive to foliage coverage or LAI which could lead to poor performance at canopy scale to monitor arsenic stress. Red edge based VIs are better in terms of sensitivity to variations in arsenic levels compared to traditional indices but still they are either sensitive to foliage cover (or LAI) (TCARI and MCARI) or both foliage cover and soil background reflectance. This study indicated that combined index, TCARI/OSAVI can resist better to LAI and soil backgrounds compared to all studied VIs, and can be useful in monitoring arsenic mitigation in contaminated rice fields through quantifying the plant stress. It is worth noting that canopy scale results are based on simulation results. In field conditions, other abiotic (e.g., atmospheric noise) and biotic (pest damage) factors confound with variations in reflectance characteristics and therefore, these relationships should also be to evaluated using field data.

## Figures and Tables

**Table 1:** Composition of the hydroponic solution used to grow rice.

Compound	Concentration
	(mM)
CaCl_2_	0.5
KNO_3_	2.0
MgSO_4_	0.5
(NH_4_)_2_SO_4_	0.5
	(µM)
FeEDTA	10.0
Na_2_MoO_4_	0.1
H_3_BO_3_	20.0
MnCl_2_	1.0
CuSO_4_	2.0
ZnSO_4_	2.0

**Table 2:** Input parameters for the Scattering by Arbitrarily Inclined Leaves (SAIL) model.

Parameter	Values
Leaf reflectance and transmittance	Four arsenic levels (*i.e.*, control, low, medium and high)
Soil reflectance	DeWitt silt loam soil (dry, wet, submerged)
Leaf area index (LAI)	0.1, 0.5, 1.0, 1.5, 2.0, 4.0, 6.0
Leaf angle distribution	Erectophile
View zenith angle	0 degrees (nadir)
Sun zenith angle	45 degrees
Fraction of direct incoming radiation	1.0

**Table 3:** Vegetative indices evaluated for prediction of As concentration.

Type	Name	Abbrev.	Equation	Reference
Red-NIR **^†^**	Normalized difference vegetation index	NDVI	(Rn − Rr)/(Rn + Rr)	[46]
Red-NIR	Optimized soil adjusted vegetation index	OSAVI	(Rn − Rr)/Rn + Rr + 0.16)	[47]
Red-RE **^‡^**	Modified chlorophyll absorption reflectance index	MCARI	[(Re − Rr) − 0.2(Re − Rg)](Re/Rr)	[31]
Red-RE	Transformed chlorophyll absorption reflectance index	TCARI	3[(Rre − Rr) − 0.2(Re − Rg)(Re/Rr)]	[48]
RE	Peaks derivative ratio	PDR	Der.720/Der.700	[45]
Combined indices	TCARI/OSAVI	-	TCARI/OSAVI	[48]

^**†**^ Near Infrared (NIR); ^**‡**^ Red Edge (RE).

**Table 4:** Performance of leaf vegetative indices to predict plant stress.

Spectral Index	Slope	Intercept	r^2^	RMSE
NDVI	−316.6	278.7	0.69	1.99
OSAVI	−74.9	44.1	0.73	1.84
MCARI	143.3	−8.6	0.85	1.23
TCARI	580.2	−31.6	0.88	1.10
PDR	−20.5	24.5	0.79	1.45
TCARI/OSAVI	163.2	−14.8	0.89	1.11

**Table 5:** Percent variation in canopy vegetative indices contributed from various factors including background (BG) reflectance, leaf arsenic concentration, LAI and their interactions.

	Source of Variation					
*Spectral Variable*	*Background*	*Arsenic Concentration*	*LAI*	*BG*x*LAI*	*BG*x*Arsenic*	*LAI*x*Arsenic*
NDVI	1.4	0.8	97.5	0.3	−	−
OSAVI	1.3	0.8	97.5	0.3	−	−
GNDVI	4.6	14.9	76.9	2.73	0.5	0.2
MCARI	0.1	45.4	51.9	−	−	2.5
TCARI	0.2	43.8	54.8	−	−	1.1
Peak Derivative Ratio	16.4	44.2	30.9	0.43	3.6	0.6
TCARI/OSAVI	2.6	74.1	22.2	0.63	0.2	0.3
Degrees of freedom	3	3	6	18	9	18

## Abbreviations

The following abbreviations are used in this manuscript:

ARS	Agricultural Research Service
As	Arsenic
ICP-AES	Inductively Coupled Plasma Atomic Emission Spectrometry
LAI	Leaf Area Index
MCARI	Modified Chlorophyll Absorption Reflectance Index
NDVI	Normalized Difference Vegetative Index
NIR	Near Infrared
NIST	National Institute of Standards and Technology
OSAVI	Optimized soil adjusted vegetation index
PPFD	Photosynthetic Photon Flux Density
SAIL	Scattering by Arbitrarily Inclined Leaves
TCARI	Transformed chlorophyll absorption reflectance index
Vis	Vegetative Indices
